# Comorbidities are associated with different features of severe asthma

**DOI:** 10.1186/s12948-018-0103-x

**Published:** 2018-12-03

**Authors:** Federica Novelli, Elena Bacci, Manuela Latorre, Veronica Seccia, Maria Laura Bartoli, Silvana Cianchetti, Federico Lorenzo Dente, Antonella Di Franco, Alessandro Celi, Pierluigi Paggiaro

**Affiliations:** 1 Department of Surgery, Medicine, Molecular Biology and Critical Care, University of Pisa, Pisa, Italy; 2 1st Otorinolaryngology Unit, University Hospital of Pisa, Pisa, Italy; 3 Cardio-Thoracic and Vascular Department, Respiratory Pathophysiology Unit, University Hospital of Pisa, Via Paradisa 2, 56124 Pisa, Italy

**Keywords:** Severe asthma, Comorbidities, Asthma control, Sputum eosinophils, Nasal polyps

## Abstract

**Background:**

According to ATS/ERS document on severe asthma (SA), the management of these patients requires the identification and proper treatment of comorbidities, which can influence the control of asthma.

**Methods:**

The aim of this study was to assess the independent effect of different comorbidities on clinical, functional and biologic features of SA. Seventy-two patients with SA according to GINA guidelines were examined. We collected demographic data, smoking habit, asthma history, and assessment of comorbidities. Pulmonary function, inflammatory biomarkers, upper airway disease evaluation, asthma control and quality of life were carefully assessed.

**Results:**

The mean age of patients was 59.1 years (65.3% female, 5.6% current smokers). Comorbidities with higher prevalence were: chronic rhinosinusitis with or without nasal polyps (CRSwNP or CRSsNP), obesity and gastro-esophageal reflux (GERD), with some overlapping among them. In an univariate analysis comparing patients with single comorbidities with the other ones, asthmatics with CRSwNP had lower lung function and higher sputum eosinophilia; obese asthmatics had worse asthma control and quality of life, and tended to have lower sputum eosinophils; asthmatics with GERD showed worse quality of life. In multivariate analysis, obesity was the only independent factor associated with poor asthma control (OR 4.9), while CRSwNP was the only independent factor associated with airway eosinophilia (OR 16.2). Lower lung function was associated with the male gender and longer duration of asthma (OR 3.9 and 5.1, respectively) and showed a trend for the association with nasal polyps (OR 2.9, p = 0.06).

**Conclusion:**

Our study suggests that coexisting comorbidities are associated with different features of SA.

**Electronic supplementary material:**

The online version of this article (10.1186/s12948-018-0103-x) contains supplementary material, which is available to authorized users.

## Introduction

Severe asthma (SA) represents a major problem in asthma management. Although severe asthma represents no more than 10% of all asthma patients, it is responsible for the large majority of direct and indirect costs for asthma [1, 2]. In Italy, the mean annual asthma-related cost of a patient with severe asthma has been estimated 3300 euros, including both direct and indirect costs [3]. Therefore, great attention has been done in the last years to a better management of this limited group of patients.

The ERS/ATS document defined SA as asthma which requires high level of inhaled therapy to be controlled or which remain uncontrolled despite that [4]. This definition includes a heterogeneous group of patients in whom the control of the disease is not achieved for different reasons [5], like a relative insensitivity to corticosteroid therapy (treatment-resistant SA) or presence of factors other than asthma, like persistent environmental exposures, psychosocial issues and comorbidities (difficult-to-treat SA), which cannot be completely removed or resolved.

The management of severe asthmatics includes a stepwise procedure in which a crucial point is the identification and proper treatment of comorbidities which can influence asthma control [6]. They are mainly represented by upper airway diseases, obesity and gastroesophageal reflux. The prevalence of these comorbidities is high in these patients [7], but there are few data on their independent effect on the different features characterizing SA, like poor control of symptoms, low lung function, airway inflammation and poor asthma related quality of life.

Aim of this study is to characterize patients with SA according to the main asthma comorbidities (in particular obesity, upper airway diseases and gastroesophageal reflux) and their independent influence on different clinical, functional and biologic features of SA. In other words, we would like to assess on which different aspects of SA (symptoms, pulmonary function, airway inflammation or quality of life) each single comorbidity may have major impact.

## Patients and methods

### Patients

We selected from our clinical routine 72 patients with asthma, who met the ERS/ATS definition of SA [4]. All of them satisfied to the following entering criteria: (a) diagnosis of asthma at the first examination in our Unit, according to GINA guidelines [8]; (b) follow-up observation of 1 year at least in our Unit, during which adherence to therapy was assessed, comorbidities were checked and appropriately treated at the best, and pharmacologic treatment was optimized; (c) persistence, at the end of this follow-up period, of current symptoms and limitation in daily life, therefore fulfitting the diagnosis of SA according to the ERS/ATS document and GINA guidelines [4, 8].

### Study protocol

All patients attended to our Unit on two different days, 4 weeks apart, in a stable phase of the disease (without asthma exacerbation in the last month), after withdrawal of pharmacological therapy in the last 24 h during which salbutamol were allowed but not, if possible, in the last 6 h.

In the first day (Visit 1) they performed, in the following order: blood analysis for white blood cells count and serum total IgE, pre and post bronchodilator spirometry, measurement of exhaled nitric oxide at a flow rate of 50 ml/s, collection of induced sputum to evaluate type and severity of airways inflammation. Patients were advised to monitor, between Visit 1 and Visit 2 during which they continued their regular treatment, the presence of day-time and night-time asthma symptoms, rescue medication use, and morning and evening Peak Expiratory Flow (PEF) using a diary card.

In the second day (Visit 2) patients performed methacoline challenge test (when possible), ENT visit with fiber-optic rhinoscopy to characterize the presence and the type of upper airways disease, in particular the presence of chronic rhinosinusitis with nasal polyps (CRSwNP) or without nasal polyps (CRSsNP). They also filled in the Asthma Control Questionnaire (ACQ) [9] and the Asthma Control Test (ACT) [10]. Quality of life was evaluated by Asthma Quality of Life Questionnaire (AQLQ) [11]. In addition, we collected for each patient demographic data, smoking habit, familiar history of asthma, age of asthma onset, anthropometric data (weight, height, body mass index (BMI)) for assess obesity (BMI ≥ 30), presence of gastroesophageal reflux disease (GERD) (relying on the presence of previously instrumental diagnosis associated with the assumption of PPI for the control of symptoms) and other important comorbidities. The number of exacerbations in the past 12 months and any hospitalization due to severe exacerbations were also recorded. Adherence to treatment was assessed by the Morisky Adherence scale [12], a four item questionnaire assessing forgetfulness, carelessness, and episodic non-adherence with medication; we considered low adherence two or more positive responses in this test.

## Methods

Pulmonary function tests were carried out on each patient by using the same equipment (Elite Series pletismography Medical Graphics, St Paul, Minnesota, USA) and made according to European Respiratory Society reference value [13, 14]. In a subset of subjects (having a baseline FEV1 greater than 1.5 L) methacholine challenge test was also performed [15].

Sputum induction and processing procedures were done according to European Respiratory Society Task Force recommendations [16], as previously described [17]. Normal values for sputum eosinophils were derived from a normal Italian population [18].

Fractional exhaled nitric oxide (FeNO) was measured at respiratory flow rate of 50 using a chemioluminescent analyzer (HypAir FeNO, Medisoft, Belgium), according to guidelines [19].

Asthma control was evaluated according to GINA guidelines [8], taking in consideration day-time and nigh-time asthma symptoms, rescue medication use, limitations in daily life in the last 4 weeks (as derived from diary card) and the presence of asthma exacerbations in the last year.

The study has been approved by the local Ethic Committee (Prot. No. 17658; March 10, 2011), and a signed informed consent was obtained by all participants.

### Statistical analysis

Data are reported as mean ± SD or median and range for continuous normally or non-normally distributed variables, and as absolute frequencies and percentages for nominal variables. Categorical variables (gender, atopy, early onset asthma, control of asthma, exacerbations, sputum eosinophilia) were compared by Chi Square analysis. Continuous data were compared using unpaired t-test or ANOVA test for age and pulmonary function, and Mann–Whitney or Kruskal–Wallis test for nonparametric data. Statistical analysis was carried out using SPSS 13 (SPSS Inc., Chicago IL, USA).

Multivariate analysis was performed, in order to assess the major determinants of some markers of severity. Dependent variables were: asthma control (not controlled vs partly or well controlled according to GINA), post-bronchodilator FEV1 (< vs ≥ 80% predicted), sputum eosinophilia (eosinophil percentage ≥ vs < 3%), and were considered as binary variables. Independent variables were: age and duration of asthma (as binary variables defined with the median value for threshold), gender (female vs male), smoking habit (current vs ex-smokers or non-smokers), CRSwNP (yes vs no), obesity (yes vs no), and GERD (yes vs no), all as binary variables.

## Results

### Descriptive characteristics of the patients

The mean age of patients was 59.1 years and 65.3% were female. Post-BD FEV1 ranged from 45 to 129% of predicted, but 61.1% of patients have normal post-BD FEV1 (> 80% of predicted). Approximately 50% of patients had poorly controlled asthma according GINA guidelines, despite the high level of asthma therapy (high dose ICS in 93.3% and medium dose in 6.9% of patients, respectively, with the addition of LABA in all patients, leukotriene receptor antagonists in 62.5%, tiotropium in 29.2%, theophylline in 19.4%, omalizumab in 18.1% and regular oral corticosteroids in 15.3%) (Table 1).

**Table 1:** Main demographic, clinical and functional data of the group of severe asthmatics

	N 72
Age, years (nean ± sd)	59.1 ± 11.1
Gender, M/F (%)	34.7/65.3
Smoke, Y/Ex/N (%)	5.5/33.3/61.2
Atopy (%)	47 (65.2)
Duration of disease, yrs (median and range)	20.6 (2–57)
Age of asthma onset, yrs (median and range)	40 (2–68)
Early onset asthma, n (%)	10 (13.8)
Post-bronc FEV1, % of pred (mean ± SD)	86.6 ± 17.8
FeNO, ppb (median and range)	22.4 (3.5–86.5)
Sputum eosinophils, % (median and range)	18 (0–95.6)
Sputum neutrophils, % (median and range)	29 (0–96.9)
Blood eosinophils, cells/µl (median and range)	285 (0–2490)
Blood Eosinophils, % (median and range)	4.5 (0–30)
Total IgE, IU/mL (median and range)	275.9 (8.1–3830)
ACT (median and range)	19 (7–25)
ACQ (median and range)	1.65 (0–3.7)
AQLQ (median and range)	4.7 (2.7–6.9)
PEF variability (median and range)	18.6 (3.7–47)
GINA, not controlled, n (%)	37 (51.4)
Exacerbations, no./last year (median and range)	1 (0–15)

*FEV1* forced expiratory volume in the first second, *PD20FEV1* provocative methacholine dose of a 20% drop in FEV1, *FeNO* fractional exhaled nitric oxide, *ACT* asthma control test, *ACQ* asthma control questionnaire, *AQLQ* asthma quality of life questionnaire

**Fig. 1:**
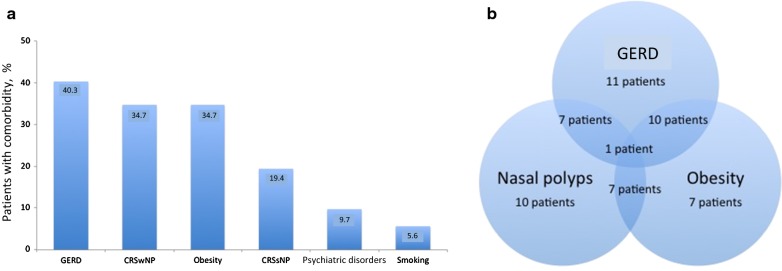
Prevalence of comorbidities (**a**) and overlap between the three most prevalent comorbidities (**b**) in the population of severe asthmatics

The large majority of the patients showed the presence of one or more comorbidities potentially affecting asthma control (Fig. 1a). No significant differences between these patients with one or more comorbidities (N = 63) or without comorbidities (N = 9) were observed for demographic, clinical, functional and biological features of the disease, except for omalizumab treatment (44% in patients without vs 14.3% with one or more comorbidities, p = 0.03). More comorbidities coexisted in the same patient (Fig. 1b).

Four patients only were current smokers. The mean packs-year of the ex-smokers was 10.6 ± 4.3. Adherence to therapy, as evaluated by Morisky questionnaire, was in general good and only the 6.9% of patients resulted poorly adherent. This may be due to the inclusion in this study of patients who had been followed in our clinic for 1 year at least, during which many strategies to optimize the adherence to therapy (use of diary, behavioral based intervention, education to medication and disease) had been used.

Considering the high prevalence of CRS, obesity and GERD, we have separately analyzed the characteristics of asthma in our patients according to the presence or absence of each single comorbidity.

### Severe asthma and single comorbidities

CRS included two different entities: CRS with nasal polyps (CRSwNP) and CRS without nasal polyps (CRSsNP) [20]. When patients with CRSwNP were compared with patients with CRSsNP, we observed the significant impact of the presence of NP on lung function, sputum eosinophilic inflammation and control of asthma (see table E1 in the Supplementary Information). According to that, we have considered for our analysis only the patients with CRSwNP.

Severe asthmatics with CRSwNP had lower lung function [FEV1: 71.1 ± 17.5 vs 80.8 ± 17.5% pred, p < 0.05] and higher sputum [31.5 (0.4–95.6) vs 8.5 (0–84.1) %, p < 0.05] and blood [6.2 (0–30) vs 3.8 (0.3–19.6) %, p < 0.05] eosinophils than asthmatics without NP. Despite this, the two groups of patients had similar indices of control and quality of life, with no difference in other clinical data. Instead, as expected, we observed a greater use of intranasal corticosteroid therapy in the group of asthmatics with NP (88% vs 63.8%, p = 0.03).

Obese asthmatics had a similar functional and inflammatory data than non-obese, but worse asthma control [ACT: 16 (7–25) vs 21 (11–25, p < 0.05] and quality of life index [AQLQ: 4.4 (3.0–6.2) vs 4.9 (2.7–6.9), p < 0.05], with no-difference in atopy, age of asthma onset, familiarity for asthma and asthma treatment. There was a trend for sputum eosinophilia to be lower in obese asthmatics [8.3 (0–71.2) vs 17.4 (0–95.6) %, p = 0.07].

The prevalence of GERD was very high. Patients with GERD showed similar demographic, functional and inflammatory characteristics, but had a worse quality of life ([QLQ: 4.4 (2.7–6.2) vs 5.0 (3.2–6.9), p = 0.04] in comparison with patients without GERD. There was no difference in prevalence of obesity between the two groups.

### Multivariate analysis of factors contributing to poor asthma control, eosinophilic airway inflammation and lower lung function

We performed multivariate analysis using the of presence of poor symptom control, or the lower lung function (Post-BD FEV1 < 80% of predicted) or the eosinophilic phenotype (sputum eosinophils ≥ 3%) as dependent variables, and age (> vs < median value 60.5 years), gender (male vs female), smoke (current smokers vs ex or nonsmokers), duration of asthma (> vs < median value 20.5 years), obesity (Y vs N), CRSwNP (Y vs N) and GERD (Y vs N) as independent variables (Table 2). Poor control of asthma symptoms was associated with obesity; airway eosinophilic inflammation was associated with CRSwNP and lower duration of asthma; and lower FEV1 was associated with longer duration of asthma, male gender and showed a trend for the association with CRSwNP.

**Table 2:** Multivariate analysis of predictors of poor control, lower lung function and sputum eosinophilia

Indipendent variables	Dependent variables OR (95% CI)		
	Poor symptom control	Lower lung function	Sputum eosinophilia ≥ 3%
Age (> vs < median value)	0.7 (0.2–1.9)	0.8 (0.3–2.5)	0.2 (0.0–1.3)
Gender (F vs M)	0.87 (0.3–2.8)	0.3 (0.1–0.9)*	1.6 (0.2–12.1)
Smoke (Yes vs No-Ex)	0.4 (0–4.9)	0.3 (0.0–4.0)	0.5 (0.0–18.7)
Duration of asthma (> vs < median value)	1.9 (0.6–6.1)	5.1 (1.4–18.8)*	0.1 (0.01–0.5)*
Obesity (Y vs N)	4.9 (1.6–15.4)*	1.6 (0.5–4.9)	0.6 (0.1–2.8)
CRSwNP (Yes vs N)	0.9 (0.3–2.7)	2.9 (1–9.1)^§^	16.2 (1.7–151.7)*
GERD (Yes vs N)	1.4 (0.5–4.2)	0.5 (0.2–1.8)	0.6 (0.1–2.9)

*CRSwP* chronic rhinosinusitis with nasal polyps, *GERD* gastro-esophageal reflux disease

* p < 0.05; ^§^ p = 0.06

## Discussion

The results of the present study confirm the high prevalence of comorbidities in patients with severe asthma, in particular obesity, CRSwNP and GERD, and show that these three major comorbidities have different impact on asthma symptom control, lower lung function and airway eosinophilic inflammation. In fact, using a multivariate analysis taking in consideration these comorbidities and age, gender, smoke and duration of asthma, obesity was the only independent factor associated with poor symptom control (OR: 4.9), while CRSwNP and duration of asthma were the only independent factors associated with airway eosinophilia (OR: 16.2 and 0.1 respectively). GERD was not associated with any specific feature of severe asthma. Lower lung function was associated with male gender and longer duration of asthma (OR 3.9 and 5.1 respectively) and showed a trend for the association with nasal polyps (OR 2.9, p = 0.06). Therefore, comorbidities may have influence on different features of severe asthma.

The prevalence of GERD and CRS in this asthmatic population is in line with previous large studies on severe asthmatics [7, 21], while the prevalence of obesity is different from other studies [22, 23], where obesity was observed up to 50% of severe asthmatics. Other comorbidities, like anxiety and depression, have been associated to a poor asthma control [24].

Previous studies have shown the association between CRS and asthma [7, 25–27], showing in particular that patients with CRSwNP had more severe asthma phenotype [28], higher eosinophilic airway inflammation [29] and a lower lung function [30]. There is now evidence that CRS with (CRSwNP) or without NP (CRSsNP) should be considered two different clinical and pathologic entities [20], with different relationship with lower airways involvement [30]. Our data confirm the close association between CRSwNP and sputum eosinophilia and lower lung function.

Obesity is another common comorbidity associated with difficult asthma: these patients show late onset asthma, frequent corticosteroid use, non-eosinophilic inflammation [7, 22, 31]. Also in our data, obese patients showed worsened control of asthma and quality of life, with a trend to have lower sputum eosinophilic inflammation.

Finally, in our sample of patients, the presence of GERD did not significantly impact on the control of asthma, airway inflammation and lung function, however resulting a significant difference in quality of life. In effect, discordant results have been produced on the impact of GERD treatment in asthma [32, 33].

The new approach reported in our study is to have considered in a multivariate analysis the major comorbidities which may affect different features of SA. We separated the presence of poor symptom control, lower lung function and eosinophilic phenotype as different characteristics of severe asthma, which are not always correlated between them. Using a multivariate analysis, we demonstrated that each comorbidity has a different impact on the various features of asthma severity. In particular, CRSwNP had an important impact on sputum eosinophilia, while obesity had a main impact on a poor control of asthma symptoms. This information confirms the large heterogeneity of SA and the complex interaction between different aspects of asthma severity.

Differently from previous studies, in our study we did not find an association between asthma comorbidities and exacerbations, while ten Brinke et al. [21] reported that recurrent exacerbations in adult SA are more frequent in patients with comorbid conditions. This difference may be due to the exclusion in that study of the patients under regular oral corticosteroid therapy, and to the fact that our patients were receiving therapy either for GERD and for CRS.

A recent paper performed on a similar sample of patients with difficult asthma reported the independent impact of some comorbidities on a broad spectrum of outcomes [34]. While similar results with our study were reported for obesity, different results were obtained for CRS and for dysfunctional breathing or vocal cord dysfunction. Differently from this study, our data reported also the impact of comorbidities on sputum eosinophilic inflammation and pulmonary function level, therefore adding new informations on this specific topic.

Considering the relevant role of comorbidities on the different features of asthma control in severe asthmatics, this study underlines the importance of a multidisciplinary approach to these severe asthmatics, requiring the collaboration of different specialties (like ENT specialist, gastro-enterologist, nutritionists, etc) coordinated in a specific patient-related journey that each tertiary center should promote.

In conclusion, our study confirms the high prevalence of comorbidities, often coexisting, in patients with SA, and highlights their association with different characteristics of the disease. This suggests the importance of the rigorous characterization of the asthmatic patients also in terms of comorbidities, for tailoring the best management. New data on this point are expected to be obtained by large national [35] and international database on severe asthma [36, 37].

## Additional file

**Additional file 1: Table S1.** Characteristics of severe asthmatics with or without nasal polyps.
