# Supplementary material for: Comorbidities are associated with different features of severe asthma
Source: Clin Mol Allergy. 2018 Dec 3;16:25. doi: 10.1186/s12948-018-0103-x (PMC6276160; doi:10.1186/s12948-018-0103-x)
Supplement: Supplementary file 1 — Additional file 1: Table S1. Characteristics of severe asthmatics with or without nasal polyps. [file 12948_2018_103_MOESM1_ESM.docx]

Table S1. Characteristics of severe asthmatics with or without nasal polyps.

|  | Asthmatics with CRSwNP  (N=25) | Asthmatics without NP  (N=47) |
| --- | --- | --- |
| Age, yrs (mean+sd) | 59.8±12.7 | 58.7±10.4 |
| Gender, M/F (%) | 40/60 | 31.9/68.1 |
| Smoke, Y/Ex/N (%) | 12/24/64 | 2.1/38.3/59.6 |
| Familiarity for asthma, n(%) | 4 (16) | 23 (50)§ |
| Pre-BD FEV1, % (mean+sd) | 71.1±17.5 | 80.8±17.5* |
| Post-BD FEV1, % (mean+sd) | 79.2±16.4 | 88.9±17.9* |
| PD20FEV1, mcg (geometric mean) | 872.2 | 453.4 |
| FeNO, ppb (median and range) | 27.5 (8.6-86.5) | 18.5 (3.5-86) |
| Sputum eosinophils, % (median and range) | 31.5 (0.4-95.6) | 8.5 (0-84.1)* |
| Sputum eosinophils ≥3%, (%) | 90 | 61.8* |
| Sputum neutrophils, % (median and range) | 19.1 (0-96.9) | 31.8 (0-95.3) |
| Blood eosinophils, % (median and range) | 6.2 (0-30) | 3.8 (0.3-19.6)* |
| ACT (median and range) | 20 (10-24) | 19.1 (7-25) |
| ACQ (median and range) | 1.8 (0.6-3.3) | 1.4 (0-3.7) |
| Poorly controlled (GINA), n(%) | 12 (48) | 25 (53.2) |
| AQLQ (median and range) | 4.6 (2.7-6.6) | 4.9 (3.0-6.9) |
| Exacerbations, no./last year (median and range) | 1 (0-6) | 1.5 (0-15) |

* p<0.05; § p<0.01

FEV1: forced expiratory volume in the first second; PD20FEV1: provocative methacholine dose of a 20% drop in FEV1; FeNO: fractional exhaled nitric oxide; ACT: asthma control test; ACQ: asthma control questionnaire; AQLQ: asthma quality of life questionnaire.
